# Cost-benefit analysis of *Chlamydia trachomatis* screening in pregnant women in a high burden setting in the United States

**DOI:** 10.1186/s12879-017-2248-5

**Published:** 2017-02-18

**Authors:** Jared Ditkowsky, Khushal H. Shah, Margaret R. Hammerschlag, Stephan Kohlhoff, Tamar A. Smith-Norowitz

**Affiliations:** Department of Pediatrics, Division of Infectious Diseases, State University of New York Downstate Medical Center, Box 49, 450 Clarkson Ave, Brooklyn, NY 11203 USA

**Keywords:** *C. trachomatis*, Chlamydia screening, Pregnant women

## Abstract

**Background:**

*Chlamydia trachomatis* is the most common bacterial sexually transmitted infection (STI) in the United States (U.S.) [1] and remains a major public health problem. We determined the cost- benefit of screening all pregnant women aged 15–24 for *Chlamydia trachomatis* infection compared with no screening.

**Methods:**

We developed a decision analysis model to estimate costs and health-related effects of screening pregnant women for *C. trachomatis* in a high burden setting (Brooklyn, NY). Outcome data was from literature for pregnant women in the 2015 US population. A virtual cohort of 6,444,686 pregnant women, followed for 1 year was utilized. Using outcomes data from the literature, we predicted the number of *C. trachomatis* cases, associated morbidity, and related costs. Two comparison arms were developed: pregnant women who received chlamydia screening, and those who did not. Costs and morbidity of a pregnant woman-infant pair with *C. trachomatis* were calculated and compared.

**Results:**

Cost and benefit of screening relied on the prevalence of *C. trachomatis*; when rates are above 16.9%, screening was proven to offer net cost savings. At a pre-screening era prevalence of 8%, a screening program has an increased expense of $124.65 million ($19.34/individual), with 328 thousand more cases of chlamydia treated, and significant reduction in morbidity. At a current estimate of prevalence, 6.7%, net expenditure for screening is $249.08 million ($38.65/individual), with 204.63 thousand cases of treated chlamydia and reduced morbidity.

**Conclusions:**

Considering a high prevalence region, prenatal screening for *C. trachomatis* resulted in increased expenditure, with a significant reduction in morbidity to woman-infant pairs. Screening programs are appropriate if the cost per individual is deemed acceptable to prevent the morbidity associated with *C. trachomatis*.

## Background

*Chlamydia trachomatis* is the most common bacterial sexually transmitted infection (STI) in the United States (U.S.) [1] and remains a major public health problem. In 2010, there were >1.3 million infections in the U.S. reported to the Centers for Disease Control (CDC) [2]. In 2013, the estimated direct lifetime cost of treatment for chlamydia and complications was > $500 million [3].

The majority of most genital chlamydia infections in women are asymptomatic [1]. Untreated infection in women can result in pelvic inflammatory disease (PID), which can cause infertility, ectopic pregnancy, and chronic pelvic pain. In addition, infants born to women with untreated chlamydial infection may acquire infection during delivery which can lead to neonatal conjunctivitis and respiratory tract infection [1]. Untreated infections in men can lead to urethritis, epididymitis, proctitis and Reiter’s syndrome [4]. Thus, screening is needed to identify and treat infections before complications develop [5].

Although several studies have investigated the cost-effectiveness of chlamydia screening for non-pregnant women [6], few studies have examined the cost-benefit of chlamydia screening in antenatal clinics [5, 7]. Currently prenatal screening for *C. trachomatis* and treatment of pregnant women is part of routine antenatal care in only a few countries globally, including the United States, Canada, Japan, and Germany [8]. One of the major reasons given for why prenatal screening has not been implemented in other countries, including the Netherlands and United Kingdom, is that it is not cost-effective [9]. Routine screening for *C. trachomatis* in pregnant women in the US was recommended by the Centers for Disease Control and Prevention in 1993 [10]. Annual screening of all sexually active women aged <25 years is recommended, as is screening of older women at increased risk for infection (e.g., those who have a new sex partner, more than one sex partner, a sex partner with concurrent partners, or a sex partner who has a sexually transmitted infection) [11]. The purpose of this study was to model the cost-benefit of *C. trachomatis* screening all pregnant women, 16–25 years of age, compared with no screening in an area with a high prevalence of chlamydia infection (Brooklyn, New York). We hypothesized that the implementation of Chlamydia screening program will avert significant morbidity, despite net cost expenditure.

## Methods

### Model parameters and primary analysis

A decision analysis model using TreeAge Prosuite 2014 software (TreeAge Software, Williamstown, MA.) was developed to assess two study arms: 1) Chlamydia screening in pregnant women and 2) No chlamydia screening in pregnant women. The model’s variables were set to mimic the characteristics of the 2015 U.S. national population for a virtual cohort of 6,444,686 pregnant women (approximate number of pregnancies in 2015) (ages 15–24). The model predicted the costs to the healthcare system and morbidity associated with screening for chlamydia over the course of 1 year. Endpoints included direct costs to health care system, rates of vertical transmission to infant, PID, spontaneous abortion associated with chlamydial infection, neonatal conjunctivitis, neonatal chlamydial pneumonia, preterm delivery and rates of treated chlamydia.

Population and disease parameters were set as point estimates, using 2015 United States Dollars (USD). The model was run using three different estimates for chlamydia prevalence in a high burden (increased chlamydial prevalence) US setting (Brooklyn, NY): 8% (pre-screening era) [12], 6.7% (current prevalence), and 16.9% (threshold prevalence at which costs associated with screening equal that of a “no screening” scenario) [12–14]. The base case analysis assumed a 100% screening rate with 98% sensitivity for chlamydia. Alternate screening rates were examined in sensitivity analyses. Rates of morbidity associated with chlamydia infection concurrent with pregnancy were set to the most recent estimates available. This study was exempt from ethical approval since we used existing data or record collection from prior literature that contained non-identifiable data about humans.

### Costs and disease parameters

The decision analysis model examined direct costs to the health-care system associated with chlamydia screening and infection during pregnancy. Costs associated with chlamydia screening were derived from current Medicaid reimbursement rates. Health-care related costs associated with morbidity were derived predominantly from previous cost-effective analysis addressing sequelae of chlamydial infection.

Chlamydia prevalence was gathered from pre-screening and current estimates of prevalence in Brooklyn, NY [12]. Rates of morbidity including vertical transmission, PID, spontaneous abortion associated with chlamydial infection, neonatal conjunctivitis, neonatal chlamydial pneumonia and preterm delivery were derived both from primary epidemiological studies [12] and previous cost-effective analyses. Costs and disease parameters are listed in Table 1.

**Table 1:** Disease epidemiological and cost data

	Value	Reference
Chlamydia Prevalence	.08/6.7	[12, 14]
NAAT chlamydia screen		
Sensitivity	0.98	[15]
Cost^a^	$33.48	Medicaid Reimbursement Rate
Chlamydia Treatment w/Azithromycin		
Success Rate	0.97	[31]
Cost ^a^	$1.86	[16]
PID		
Prevalence	0.27	[32]
Cost	$10,420	[8, 28]
Miscarriage		
Prevalence	0.079	[33]
Cost	$1,000	[34]
Preterm Delivery		
Prevalence	0.007	[35]
Cost ^a^	$51,589	[36]
Vertical Transmission		
Prevalence	0.46	[12]
Neonatal Conjunctivitis		
Prevalence	0.41	[12]
Cost ^a^	$83.23	[5]
Neonatal pneumonia		
Prevalence	0.16	[12]
Cost ^a^	$577.11	[5]
Infertility		
Infertility associated with Chlamydia	.00845	[37]
Infertility associated with PID	.123	[37]
Prevalence	0.33	[38]
Cost ^a^	$6060.82	[39, 40]

Values and costs associated with each variable in the model

*NAAT* nucleic acid amplification test, *PID* pelvic inflammatory disease

^a^Costs expressed in 2015 USD

### Epidemiological model

**Fig. 1:**
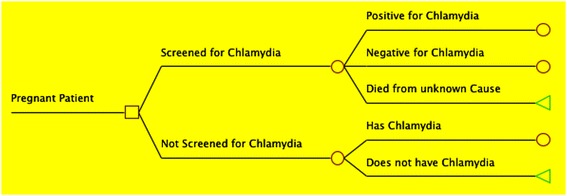
Decision Tree Study Arms: Sample of decision tree displaying the two study arms, slightly modified: A scenario in which pregnant women are screened for chlamydia, and a scenario in which no screening program exists. *Square*: origin node; *Circle*: chance node; *Triangle*: terminal node

**Fig. 2:**
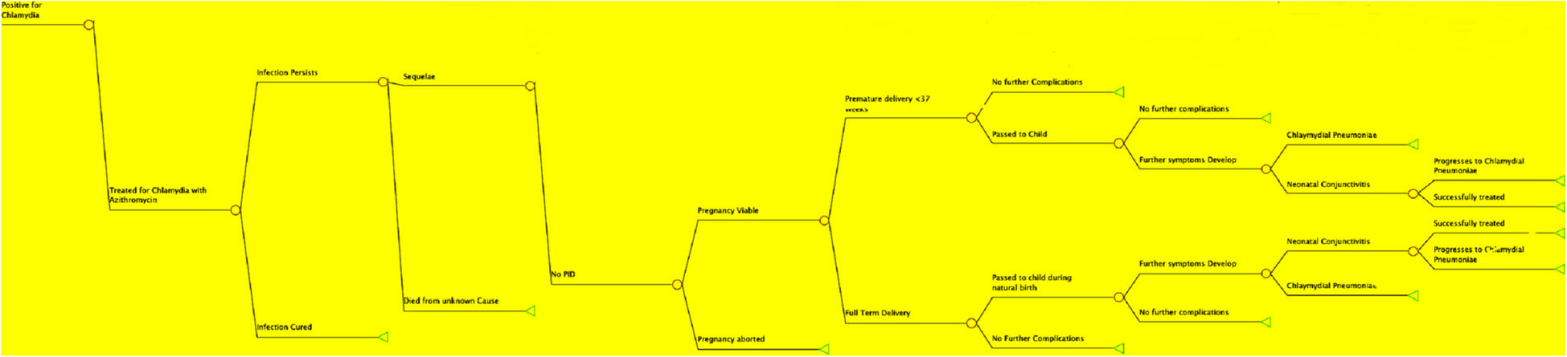
Sample of Post-Chlamydia Infection Decision Tree: Figure displays a simplified breakdown of the decision tree used to determine morbidity and costs associated with chlamydia in a pregnant woman *Circle*: chance node; *Triangle*: terminal node

Identical cohorts of pregnant women were placed into a “Screening” or “No Screening” group. Individuals could either be chlamydia positive or negative. Those in the “Screening” scenario either received or forewent screening, with a sensitivity of 98% for those screened [15]. Chlamydia positive individuals in either group could be asymptomatic, treated, or lost to follow up. If lost to follow-up, or with treatment failure, individuals could receive no sequelae, or enter a morbidity branch including vertical transmission, PID, spontaneous abortion endpoints could exist in a single individual. As hypothetical cohort members entered different branches, they accrued costs associated with screening and each aforementioned endpoint. Rates of morbidity and costs to the health-care system were recorded as they were accumulated. Samples from the decision analysis tree are presented in Figs. 1 and 2.

### Primary analysis

The primary analysis considered a pre-screening prevalence of 8% with an assumed screening rate of 100% [12]. It predicted absolute and net costs to the healthcare system, as well as morbidity in either scenario. The analysis was conducted with a post-screening era modern prevalence of 6.7% [14]. Additionally, a threshold analysis that examined outcomes at a prevalence in which costs associated with screening equaled that of a “No Screening” scenario was performed.

### Sensitivity analysis

Sensitivity analyses were conducted for all variables present in the model, varied across published ranges or set to +/-50% of the base case parameter. In-depth analyses were performed for key variables including rates of untreated chlamydia, cost of chlamydia screening, and rates of screening coverage. A multiway probabilistic analysis, in which those three variables were simultaneously varied across a certain distribution, was conducted. These variables were distributed across a triangular distribution, with the base case being most likely and the end of the ranges set to +/-50% of the base variable. The distribution for screening coverage was set between 50–100%. The multiway probabilistic analysis consisted of 100 runs and 100,000 μ simulations. Sensitivity analyses were conducted with a base chlamydia pre-screening prevalence of 8%.

## Results

### Primary analysis

Considering a cohort of 6,444,686 pregnant women in the 2015 US population in the base case analysis of a 100% screening rate with 8% prevalence of chlamydia [12], the model estimated a screening program would cost $256.305 million dollars per year to the healthcare system including screening and treatment expenses, and result in 496,000 treated cases of chlamydia. In a “No Screening” scenario, there is an estimated cost of $131.655 million, result in 168,000 treated cases of chlamydia. Ultimately, the model estimates that a screening program would result in an increased expense of $124.65 million, with 328,000 more cases of chlamydia treated. This calculates out to $19.34 per screened individual. Other estimated morbidities are shown in Table 2.

**Table 2:** Primary analysis

	Screened for *Chlamydia*	No screening
8% Prevalence		
Cost^§^	256,305,162	131,664,935
Treated Chlamydia	496,241	167,562
PID	6393	23,658
SAB	3	10
Vertical Transmission	8211	37,050
Neonatal conjunctivitis	6806	29,755
Pneumonia	3093	13,585
Premature	3	6
Infertility	786	2910
WTP:	19.34	---
6.7% Prevalence		
Cost	249,087,114	106,421,100
Treated Chlamydia	204,632	66,819
PID	2673	9852
SAB	3	9
Vertical Transmission	4999	15,427
Neonatal conjunctivitis	4101	12,386
Pneumonia	1863	5659
Premature	2	1
Infertility	329	1222
WTP:	22.14	---
Threshhold-.169 prevalence		
Cost^§^	*279,158,019*	*279,151,574*
Treated Chlamydia	1,044,039	348,013
PID	6528	49,985
SAB	6	21
Vertical transmission	9757	78,264
Neonatal conjunctivitis	8301	62,855
Pneumonia	3738	28,718
Premature	5	19
Infertility	810	6198
WTP:	0	---

*PID* pelvic inflammatory disease, *SAB* spontaneous abortion, *WTP* willingness to pay

^§^2015 USD

Using a post-screening modern prevalence estimate of 6.7% [13], there was an estimated net increase in expenditure of $142.660 million, with 204,630 cases of treated chlamydia. This leads to $22.14 per screened individual. A threshold analysis was conducted that examined the prevalence at which the cost of screening equaled the costs averted, a prevalence of 16.9%. At this prevalence, the cost associated with both scenarios is $279.158 million, with 696,000 treated cases of chlamydia. All other estimates of morbidity are shown in Table 2.

### Sensitivity analysis

Parameters affecting the health-related outcomes and costs associated with chlamydia in a screening and non-screening scenario were varied, either by +/-50% of their base value, across published ranges, or to encompass a wide variety of values. All variables were tested, and those with significant impact on outcomes are reported. In all situations, the cost of screening outweighed the cost of no screening, but significant morbidity was consistently averted (Table 3). When the cost of chlamydia screening was reduced by 50%, net cost expenditure was lowest, at $16.4 million, $2.55 per screened individual.

**Table 3:** Sensitivity analysis

	Screening		No Screening	
Treatment Rate w/o Screening	−50%	(±)50%	−50%	(±)50%
Cost^a^	256,369,609	256,047,375	154,543,570	109,301,875
SAB	3	2	5	15
Pneumonia	3106	3061	16,769	10,486
PID	6574	5298	29,194	18,258
Neonatal Conjunctivitis	7321	6284	36,702	22,969
Vertical Transmission	9564	7495	45,777	28,537
Treated Chlamydia	470,462	541,354	83,781	244,898
Premature	3	2	7	4
Infertility	815	657	3620	2264
Cost of Chlamydia Screening				
Cost^a^	148,098,884	363,222,503	131,664,935	131,664,935

Outcomes of all endpoints when the values of high impact variables were varied over a range of +/-50% of base value

*SAB* spontaneous abortion, *PID* pelvic inflammatory disease

^a^Costs expressed in 2015 USD

The percent of individuals screened for chlamydia was varied between 60–100%, in 10% intervals, as part of the sensitivity analysis, with 100% acting as the primary analysis reported above. When decreased to 60%, net cost expenditures were $51.235 million. Significant morbidity associated with the model’s endpoints was still averted, as reported in Table 4.

**Table 4:** Screening coverage rate

	100% (base case analysis)	90%	80%	70%	60%
Cost ^a^	256,305,162	245,671,430	221,104,287	207,838,030	182,900189
SAB	3	32	38	49	54
Pneumonia	3,093	3,351	4,021	5,027	5,530
PID	6,393	6,896	7,585	8,344	9,467
Neonatal Conjunctivitis	6,806	7,366	8,471	9,318	10,996
Vertical Transmission	8,211	8,533	9,642	10,563	12,042
Treated Chlamydia	496,241	431,794	362,707	333,690	296,941
Premature	3	28	31	36	43
Infertility	380	841	925	1,111	1,155

Outcomes of all endpoints when the % of total women screened by program was varied between 60–100%

*SAB* spontaneous abortion, *PID* pelvic inflammatory disease

^a^Costs expressed in 2015 USD

### Monte Carlo multiway probabilistic analysis

**Fig. 3:**
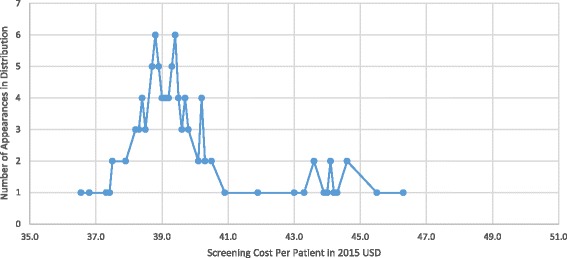
Monte Carlo Probabilistic Sensitivity Analysis: Figure shows number of times a certain cost per individual appeared in 100 iterations of 10,000 μ-simulations during the probabilistic sensitivity analysis when high impact variables were simultaneously randomly distributed across specific ranges

A Monte Carlo multiway probabilistic sensitivity analysis was conducted to offer end-point variable precision similar to a true approximate randomization test, and to offer an efficient implementation of hypothesis tests. In this analysis four variables with the greatest impact on outcomes were varied according to a triangular distribution of +/-50%, or across the range used in the one-way sensitivity analysis. These included: 1) probability of receiving chlamydia treatment without screening, 2) percent of population covered by screening 3) the cost of chlamydia screening and 4) chlamydia prevalence. The absolute cost of a screening program was then tested. The median cost was $254.565 million associated with a screening program, with a mean of $243.958 million. The interquartile range was $228.127 million - $260.439 million. There were no iterations out of the 100 run in which costs associated with a screening program were lower than that of a non-screening scenario (Fig. 3).

## Discussion

Based on our decision analysis model, the total annual health care cost associated with chlamydia is greater when a screening program is implemented in both high and low burden settings. However, there is a significant decrease in chlamydia associated morbidity, which may offset the increased cost of screening given an appropriate willingness-to-pay (WTP). These results are consistent with several prior cost-effective models that show increased cost of chlamydia screening, often offset by quality adjusted life (QALY) units and monetary conversion, with a notable decrease in associated morbidity [3, 5, 16, 17]. Current US guidelines recommend screening for chlamydia in early pregnancy despite a distinct lack of evidence as to outcomes [18]. Since these recommendations were set knowing there is likely no net cost savings associated with chlamydia screening, the averted morbidity reported is of primary importance.

### Main findings

The findings from the current investigation may offer guidance as to the future recommendations for chlamydia screening in both high and low burden settings. Despite increased cost expenditures, the cost thresh hold necessary to implement a program is low per individual, at $19.34 considering an 8% prevalence rate. The suggestion that this is a low cost per individual is pronounced when compared values for other STI screening recommendations such as that for Hepatitis B, which is estimated to cost $75.45 per individual when screening pregnant women in the US [19]. It is important to mention that in this study, screening was shown to significantly decrease major chronic sequelae of chlamydia such as infertility. Rates decreased by almost 400%, which translates to large absolute numbers, particularly in high burden populations. This can be further extrapolated to account for QALYs saved for neonates when chlamydia-associated infertility and subsequent inability to carry a pregnancy is averted. Consequently, concerns regarding the cost associated with chlamydia screening for pregnant women, particularly as they apply to high burden settings, may be abated by monetary values revealed in this model when compared to other accepted programs.

Previous studies in our laboratory examined incidence and treatment outcomes of chlamydial conjunctivitis in the prescreening era offer support for our model’s results. Hammerschlag, et al. [12] reported in a vertical transmission data study that prenatal screening and treatment of pregnant women was the most effective way to prevent neonatal chlamydial infections, especially as neonatal ocular prophylaxis has not been demonstrated to be effective in prevention of neonatal chlamydia conjunctivitis [12, 20]. These results may also be relevant to other endpoints found in our model, and offers support for the importance of a screening program by reducing neonatal chlamydia-associated morbidity, as evidenced in the present study [12].

Ong, et al [5], in a study from Australia, found that antenatal screening of women aged 16–25 is likely to be cost-effective with significantly reduced morbidity. The findings from our current decision analysis model are in agreement with the aforementioned studies. Furthermore, Ong, et al [5] agree with the current analysis that although there is net cost expenditure at predicted levels of chlamydia prevalence, infection rates rise cost savings quickly outpace expenditures [5]. These results hold true despite using prevalence and vertical transmission data gathered through two different methodologies. Ong et al [5] reported prevalence rates that were gathered from Australian family planning clinic records, and vertical transmission data was gathered from previously published studies in other populations [5]. The prevalence rates used in the current study were based on screening of over 4,000 pregnant women in one medical center in central Brooklyn [12]. Furthermore, vertical transmission data was based on screening 4357 pregnant women for cervical chlamydial infection, of whom 341 (8%) had positive cultures [12]; 230 of their infants were for followed for 3 months and evaluated for development of neonatal chlamydial conjunctivitis, pneumonia and nasopharyngeal infection with serial cultures. Additionally, the rate of chlamydia infection among women less than 18 years of age was 14% [12]. Despite values for these important variables coming from two different methodologies (Ong et al. [5] and direct screening in the current study), the results remained grossly similar notwithstanding expected differences in exact numbers and ranges.

In the current investigation the increased cost associated with screening as determined by our model does not consider the long-term effect of decreasing prevalence of chlamydia in a population. Through comparison of results using the pre-screening prevalence of chlamydia (8%) to those of the post-screening modern era (6.7%), it appears as though a modern screening program results in a lower decrease in chlamydia-associated morbidity with a large increase in net cost, as compared to a non-screening scenario [21]. Although these results are accurate for areas with a similar or otherwise low burden prevalence rate that have yet to implement a screening program, or implemented one despite a low prevalence rate, this does not hold true for areas with a high prevalence prescreening. In a situation with a high prevalence pre-screening that has, as a consequence of screening, decreased its prevalence rate, there is significant cost savings and decline in morbidity associated with a decrease in prevalence rates. As such, the estimates revealed in this model likely underestimate true cost savings and morbidity decline associated with the long-term implementation of a chlamydia screening program [21].

Currently, there are no systemic review studies that have investigated the effect of chlamydia screening specifically in pregnant women. However, the United States Preventive Services Task Force (USPSTF) recommends screening for chlamydia in early pregnancy. Current guidelines also recommend screening in early pregnancy for other sexually transmitted infections (STIs) including HIV, gonorrhea and Hepatitis B [18]. Similarly, little evidence exists for clinical outcomes associated with screening for these STIs, particularly in pregnancy. However given the low harm, high potential benefit and relatively low cost associated with screening for these STIs, as evidence by this model and other similar models, early screening for STIs during pregnancy has been determined to be an acceptable use of healthcare resources [18].

Prenatal screening and treatment is more effective than other control methods, specifically, neonatal ocular prophylaxis. Hammerschlag, et al [12] demonstrated that neonatal ocular prophylaxis with erythromycin or tetracycline ophthalmic ointments was ineffective for prevention of chlamydial ophthalmia in infants, as well as having no effect on respiratory infection [12]. The Canadian Pediatric Society recently recommended that neonatal ocular prophylaxis for *C. trachomatis* infection be stopped, and emphasized the importance of prenatal screening and treatment [22, 23]; Currently, the American Academy of Pediatrics (AAP) is considering a program similar to that in Canada. Prenatal screening may be an important public health intervention in low resource areas, especially when, affordable, sensitive NAAT based screening tests become available. It should also be mentioned that some countries that currently do not screen actually have high prevalence of *C. trachomatis* infection (>8%) in some of their populations, including the Netherlands (8%) [24] and Ireland (5.6% overall, 9.1% in women 16–18 years of age) [25].

Several advantages to expanding or initiating chlamydia screening programs outside of averted morbidity exist. It is possible, that with increased screening a higher burden of chlamydia in the population will be discovered, revealing the program to be increasingly cost-effective. Furthermore, since programs can be rolled out into already present antenatal care schedules, there is a decreased cost associated with administration, as well as decreased indirect costs to the patient. Sexual histories can be unreliable when determining the clinical risk of chlamydia infection, an opt-out approach to screening can avoid this uncertainty altogether [5]. Finally, identification of an infected mother offers the opportunity to provide treatment for sexual partner to help prevent re-infection and chlamydia-associated morbidity in the partner.

### Strengths and limitations

Strengths of this study included our ability to base our cost estimates and disease parameters from published studies without assumptions and with few calculated estimates. Those estimates that were calculated utilized linear regression between data points or simple algebra. Most epidemiological and cost data came from U.S. sources, adding to the accuracy of the results. Additionally, using data available in the literature, we were able to examine numerous endpoints associated with the health of both the pregnant woman and fetus/newborn. We were able to focus our study on a high burden setting and examine the effect of a screening program at two point prevalences: at implementation, and many years later after a screening program has been able to affect infection rate. Finally, it should be mentioned that given the accuracy of the data based in strong epidemiology, and robustness of our results in the setting of sensitivity analyses, this study is generalizable to other settings with a variety of chlamydia prevalence. The exception is that healthcare costs may differ between settings, which should be considered when applying these results to another location.

In addition to its strengths, several limitations to the study should be mentioned. We utilized a static decision analysis model that only accounted for 1 year of data. This was done to avoid complications associated with following the outcomes of pregnant women screened for chlamydia after the pregnancy has ended, and risks to the newborn and mother are greatly modified. It does, however, preclude a dynamic study of infection, which would account for changing prevalence and infection rates as the modeled screening program progresses. Additionally, as noted in previous studies, one aspect of a screening program that leads to greater cost-effectiveness is the ability to treat partners and subsequent decreased risk of reinfection. Due to a paucity of data regarding rates of partner treatment as well as asymptomatic chlamydial treatment, we were unable to incorporate this variable into the model. The risk of acquiring chlamydia-related sequelae is applied to all cases of chlamydia at the time of infection and does not account for the duration of the infection. Finally, there exists uncertainty regarding the estimated rates of chlamydia-related sequelae, which may lead to an overestimation of the cost-savings associated with a chlamydia screening program.

### Interpretation

Increasingly accurate cost-benefit models are necessary for properly evaluating the development, implementation and maintenance of screening programs. To more accurately develop screening programs associated with chlamydia more epidemiological data should be gathered regarding three major variables that, if proper data existed, would have improved the robustness of this model’s results. These are the percent of chlamydia infection that goes untreated, rates of asymptomatic chlamydia and rates of partner treatment coverage as a result of screening. The addition of the variables to the model would result in more accurate results, and the model likely would have predicted increased cost savings and averted morbidity associated with screening than was presented. Future studies should focus on these epidemiological parameters.

Prior literature has reported that in low resource countries with high chlamydia prevalence, such as Papua New Guinea and many African countries where prevalence of infection in pregnant women may exceed 20%, increased expenditures may be of particular concern [26]. Implementation of a screening program may prove particularly beneficial within a few years. Single dose treatment with azithromycin is the current standard of care [27]. The development of sensitive and affordable point of care nucleic acid amplification tests (NAAT) will make testing feasible in these regions [28]. This will help further offset costs associated with screening, and may even lead to net cost savings. Studies from Zambia show that an integrated approach to antenatal screening that builds on existing programs, such as those targeting HIV prevention, can help manage both STI infections in pregnancy, and help to overall increase antenatal attendance. This will help further offset costs associated with screening, especially when building a screening program based on pre-existing programs, and may even lead to net cost saving [8].

## Conclusion

In summary, our finding and those of previous studies support consideration that Chlamydia screening does result in net expenditure, though with a significant amount of averted morbidity [5, 8, 16, 21, 29],. Currently, the WHO does not recommend prenatal screening for chlamydia [30]. The data presented here can be utilized by policy makers and public health researchers to appropriately distribute funding and resources towards where it would most benefit the target population.

## Abbreviations

CDC: Centers for Disease Control and Prevention
NAAT: Nucleic acid amplification tests
PID: Pelvic inflammatory disease
QALY: Quality adjusted life year
STI: Sexually transmitted infections
US: United States
USD: United States dollars
USPSTF: United States Preventive Services Task Force
WTP: Willingness to pay
